# Dichlorido[1-(2-methyl­benz­yl)-3-(η^6^-2,4,6-trimethyl­benz­yl)-1*H*-2,3-dihydro­benzimidazol-2-yl­idene]ruthenium(II) dichloro­methane solvate

**DOI:** 10.1107/S160053680900350X

**Published:** 2009-02-04

**Authors:** Hakan Arslan, Don VanDerveer, Sedat Yaşar, İsmail Özdemir, Bekir Çetinkaya

**Affiliations:** a Department of Natural Sciences, Fayetteville State University, NC 28301, USA; b Department of Chemistry, Faculty of Pharmacy, Mersin University, Mersin TR 33169, Turkey; c Department of Chemistry, Clemson University, SC 29634, USA; d Department of Chemistry, Faculty of Sciences and Arts, Inönü University, Malatya TR 44280, Turkey; e Department of Chemistry, Faculty of Science, Ege University, Bornova-Izmir TR 35100, Turkey

## Abstract

The title complex, [RuCl_2_(C_25_H_26_N_2_)]·CH_2_Cl_2_, is best thought of as containing an octa­hedrally coordinated Ru center with the arene occupying three sites. Two Ru—Cl bonds and one Ru–carbene bond complete the distorted octa­hedron. The carbene portion of the ligand is a benzimidazole ring. This ring is connected to the C_6_H_2_(CH_3_)_3_ arene group by a CH_2_ bridge. This leads to a system with very little apparent strain. A dichloro­methane solvent mol­ecule completes the crystal structure. Further stabilization is accomplished *via* C—H⋯N and C—H⋯Cl interactions.

## Related literature

For synthesis, see: Yaşar *et al.* (2008 ▶); Çetinkaya *et al.* (2001 ▶, 2003 ▶); Özdemir *et al.* (2001 ▶, 2004 ▶). For general background, see: Herrmann (2002 ▶); Herrmann *et al.* (1995 ▶); Navarro *et al.* (2006 ▶); Arduengo & Krafczyc (1998 ▶). For related compounds, see: Begley *et al.* (1991 ▶); Steedman & Burrell (1997 ▶); Arslan *et al.* (2004 ▶, 2005 ▶, 2007 ▶).
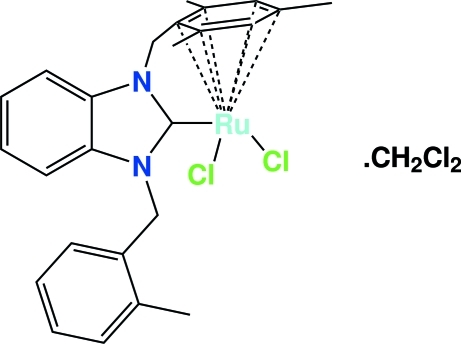


## Experimental

### 

#### Crystal data

[RuCl_2_(C_25_H_26_N_2_)]·CH_2_Cl_2_
*M* *_r_* = 611.37
Monoclinic, 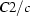

*a* = 31.362 (6) Å
*b* = 8.1014 (16) Å
*c* = 20.484 (4) Å
β = 100.11 (3)°
*V* = 5123.8 (18) Å^3^
*Z* = 8
Mo *K*α radiation
μ = 1.05 mm^−1^
*T* = 153 (2) K
0.46 × 0.14 × 0.06 mm

#### Data collection

Rigaku Mercury CCD diffractometer
Absorption correction: multi-scan (REQAB; Jacobson, 1998 ▶) *T*_min_ = 0.644, *T*_max_ = 0.940
15807 measured reflections
4501 independent reflections
3825 reflections with *I* > 2σ(*I*)
*R*_int_ = 0.054

#### Refinement

*R*[*F* ^2^ > 2σ(*F* ^2^)] = 0.049
*wR*(*F* ^2^) = 0.115
*S* = 1.08
4501 reflections
298 parameters
H-atom parameters constrained
Δρ_max_ = 0.92 e Å^−3^
Δρ_min_ = −0.73 e Å^−3^

### 

Data collection: *CrystalClear* (Rigaku/MSC, 2006 ▶); cell refinement: *CrystalClear*; data reduction: *CrystalClear*; program(s) used to solve structure: *SHELXTL* (Sheldrick, 2008 ▶); program(s) used to refine structure: *SHELXTL*; molecular graphics: *SHELXTL*; software used to prepare material for publication: *SHELXTL*.

## Supplementary Material

Crystal structure: contains datablocks I, global. DOI: 10.1107/S160053680900350X/at2716sup1.cif

Structure factors: contains datablocks I. DOI: 10.1107/S160053680900350X/at2716Isup2.hkl

Additional supplementary materials:  crystallographic information; 3D view; checkCIF report

## Figures and Tables

**Table 1:** Hydrogen-bond geometry (Å, °)

*D*—H⋯*A*	*D*—H	H⋯*A*	*D*⋯*A*	*D*—H⋯*A*
C15—H15*C*⋯N2	0.98	2.60	3.244 (7)	123
C18—H18*A*⋯Cl2	0.99	2.67	3.468 (5)	138
C23—H23*A*⋯Cl1^i^	0.95	2.78	3.730 (5)	175
C26—H26*A*⋯Cl2^ii^	0.99	2.46	3.431 (6)	168

Symmetry codes: (i) 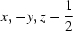; (ii) 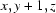.

**Fig. 1.**
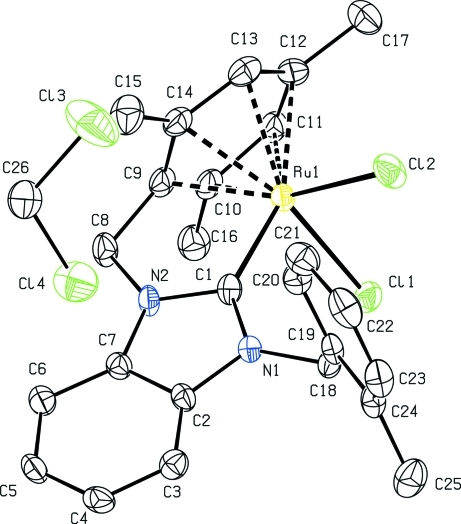
The molecular structure of the title compound, showing the atom-numbering scheme and displacement ellipsoids drawn at the 50% probability level.

**Fig. 2.**
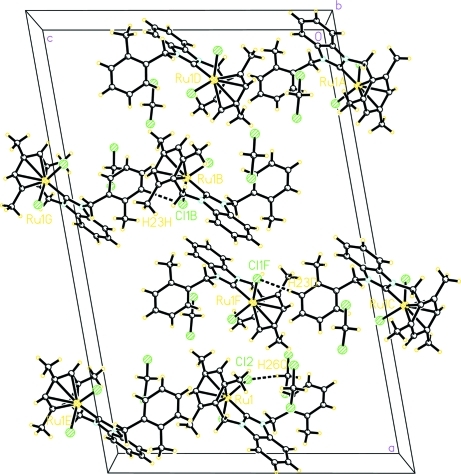
A packing diagram for (I). [Symmetry codes: A = 1 - x, y, 0.5 - z; B = -1/2 + x, 1/2 + y, z; C = 1.5 - x, 1/2 + y, 0.5 - z; D = 1 - x, 1 - y, 1 - z; E = x, 1 - y, 1/2 + z; F = 1.5 - x, 0.5 - y, 1 - z; G = -1/2 + x, 0.5 - y, 1/2 + z; H = x, -1 + y, z ].

**Fig. 3.**
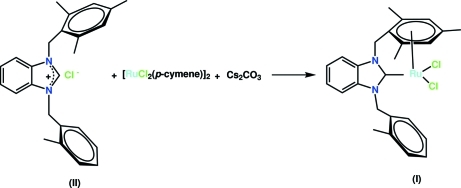
Synthesis of Ru(NHC) complex.

## supplementary crystallographic information

### Comment

The ruthenium complexes of *N*-Heterocyclic carbenes have proved to be excellent catalysts for the Suzuki-Miyura, Sonogashira, Stille and Heck reactions (Herrmann *et al.*, 1995; Herrmann, 2002; Navarro *et al.*, 2006; Arduengo & Krafczyc, 1998). Expressive examples are found in various catalytic reactions with ruthenium catalysts for alken metathesis, cycloisomerization, and cyclopropanation reactions (Özdemir *et al.*, 2004).

Previous work from our research groups in this area has focused on the elaboration of olefins as electron-rich heterocyclic carbene precursors which allow the formation of chelating carbenes, on the rapidly developing chemistry of *η*^6^-arene ruthenium(II) complexes containing substituted imidazolidin-2-ylidenes (Özdemir *et al.*, 2001; Çetinkaya *et al.*, 2001, 2003), and on the synthesis, characterization and uses of palladium, platinum and ruthenium *N*-heterocyclic carbene complexes as catalysts (Yaşar *et al.*, 2008; Arslan *et al.*, 2004, 2005, 2007, and references therein).

In the present study, we have synthesized and characterized a new ruthenium complex, (1-(2-methylbenzyl)-3-(2,4,6-trimethylbenzyl)-1*H*-benzo[*d*]imidazol-2(3*H*)-ylidene)ruthenium(II) dichloride. dichloromethane solvate, (I). The crystal structure of the title compound, (I), is depicted in Fig. 1.

The benzimidazol ring which has a carbene portion is connected to the C_6_H_2_(CH_3_)_3 _arene by a CH_2_ bridge. This leads to a system with very little apparent strain. The ruthenium atom in the title compound is best described as having an octahedral coordination environment, with the arene occupying three coordination sites. Two coordination sites are occupied by Cl ligands, while the sixth site is occupied by the carbene carbon of the benzimidazol ring.

The ruthenium atom is situated 1.6766 (19) Å from the ring centroid of the arene. While there are substantial differences in the C—C and Ru—C distances [Ru—C 92.099 (5), –C10 2.161 (4), –C11 2.246 (4), –C12 2.282 (5), –C13 2.203 (5), –C14 2.198 (5) Å] for the arene ring, there is no evidence of the alternating C—C bonds observed in some ruthenium-arene complexes (Begley *et al.*, 1991).

The arene, the 2-methylbenzyl, imidazol and benzimidazol rings are almost planar with a maximum deviation of 0.038 (5) Å for atom C14, 0.015 (5) Å for atom C21, 0.004 (4) Å for atom N2, and 0.023 (5) Å for atom C5. The five-membered imidazole ring forms dihedral angles of 87.30 (4) ° and 78.53 (4) ° with the 2-methylbenzyl and 2,4,6-trimethylbenzyl rings, respectively.

The small steric demand of the benzimidazole ligand is reflected in the Cl—Ru—C1 angles, which are 87.51 (12) ° and 97.42 (12) °. These are significantly larger than the angles in the pyridine substituted complexes [RuCl_2_(py)(η^6^-arene)] (Steedman & Burrell, 1997), and agree with Arslan results, (Arslan *et al.*, 2004, 2005, 2007, and references therein). On the other side, the Ru—Cl distances in the coordination sphere are equal within experimental error [Ru—Cl1 = 2.4167 (12) Å and Ru—Cl2 = 2.4175 (13) Å]. The Cl—Ru—Cl angle is 88.52 (5) °.

The components of the title compound are assembled by two intermolecular C—H···Cl hydrogen bonds, to form a three-dimensional framework (Fig. 2 and Table 1). The intramolecular contacts, C—H···N and C—H···Cl, are also listed in Table 1.

### Experimental

A suspension of 1-(2-methylbenzyl)-3-(2,4,6-trimethylbenzyl)benzimidazolium chloride (1.00 g, 2.56 mmol), Cs_2_CO_3 _(0.84 g, 2.56 mmol), [RuCl_2_(*p*-cymene)]_2 _(0.78 g, 1.28 mmol) and molecular sieves was heated under reflux in degassed dry toluene (20 ml) for 12 h. The reaction mixture was then filtered while hot, and the volume was reduced to about 10 ml before addition of *n*-hexane (10 ml). The precipitate formed was crystallized from CH_2_Cl_2_:hexane (5:10 ml) to give crystal product (Figure 3). Yield 0.58 g (86%), *M*.p.: 549–550 K. FT—IR (KBr pellet, cm^-1^): ν_CN_ 1424 cm^-1. 1^H NMR (δ, 399.9 MHz, CDCl_3_): 2.18 and 2.34 [s, 9H, CH_2_C_6_H_2_(C*H*_3_)_3_-2,4,6]; 2.39 [s, 3H, CH_2_C_6_H_4_(C*H*_3_)-2]; 5.08 [s, 2H, C*H*_2_C_6_H_4_(CH_3_)-2]; 5.59 [s, 2H, C*H*_2_C_6_H_2_(CH_3_)_3_-2,4,6]; 5.76 [s, 2H, CH_2_C_6_*H*_2_(CH_3_)_3_-2,4,6]; 6.80–7.50 [m, 8H, NC_6_*H*_4_N and CH_2_C_6_*H*_4_(CH_3_)-2]. ^13^C{H} NMR (δ, 100.5 MHz, CDCl_3_): 17.0 and 17.4 [CH_2_C_6_H_2_(*C*H_3_)_3_-2,4,6]; 19.5 [CH_2_C_6_H_4_(*C*H_3_)-2]; 49.7 [*C*H_2_C_6_H_2_(CH_3_)_3_-2,4,6]; 53.5 [*C*H_2_C_6_H_4_(CH_3_)-2]; 90.0, 92.8, 98.6, 101.6, 110.0, 112.5, 123.4, 123.8, 126.0, 127.0, 127.1, 130.2, 133.2, 134.8, 135.0 and 135.4 [CH_2_*C*_6_H_2_(CH_3_)_3_-2,4,6; N*C*_6_H_4_N and CH_2_*C*_6_H_4_(CH_3_)-2]; 185.9 [*C*_carbene_].

### Refinement

H atoms were geometrically fixed and allowed to ride on the parent atom with C—H = 0.95 - 0.99 Å, and *U*_iso_(H) = 1.5*U*_eq_(C) for methyl H atoms and 1.2*U*_eq_(C) for other H atoms.

### Crystal data

**Table:**

[RuCl_2_(C_25_H_26_N_2_)]·CH_2_Cl_2_	*F*(000) = 2480
*M**_r_* = 611.37	*D*_x_ = 1.585 Mg m^−^^3^
Monoclinic, *C*2/*c*	Mo *K*α radiation, λ = 0.71073 Å
Hall symbol: -C 2yc	Cell parameters from 5683 reflections
*a* = 31.362 (6) Å	θ = 2.8–26.0°
*b* = 8.1014 (16) Å	µ = 1.05 mm^−^^1^
*c* = 20.484 (4) Å	*T* = 153 K
β = 100.11 (3)°	Rod, orange
*V* = 5123.8 (18) Å^3^	0.46 × 0.14 × 0.06 mm
*Z* = 8	

### Data collection

**Table:**

Rigaku Mercury CCD (2x2 bin mode) diffractometer	4501 independent reflections
Radiation source: Sealed Tube	3825 reflections with *I* > 2σ(*I*)
Graphite Monochromator	*R*_int_ = 0.054
Detector resolution: 14.6306 pixels mm^-1^	θ_max_ = 25.2°, θ_min_ = 2.8°
ω scans	*h* = −28→37
Absorption correction: multi-scan (REQAB; Jacobson, 1998)	*k* = −9→9
*T*_min_ = 0.644, *T*_max_ = 0.940	*l* = −23→24
15807 measured reflections	

### Refinement

**Table:**

Refinement on *F*^2^	Primary atom site location: structure-invariant direct methods
Least-squares matrix: full	Secondary atom site location: difference Fourier map
*R*[*F*^2^ > 2σ(*F*^2^)] = 0.049	Hydrogen site location: inferred from neighbouring sites
*wR*(*F*^2^) = 0.115	H-atom parameters constrained
*S* = 1.08	*w* = 1/[σ^2^(*F*_o_^2^) + (0.0431*P*)^2^ + 37.6344*P*] where *P* = (*F*_o_^2^ + 2*F*_c_^2^)/3
4501 reflections	(Δ/σ)_max_ = 0.001
298 parameters	Δρ_max_ = 0.92 e Å^−^^3^
0 restraints	Δρ_min_ = −0.73 e Å^−^^3^

### Special details

**Table:**

Geometry. All e.s.d.'s (except the e.s.d. in the dihedral angle between two l.s. planes) are estimated using the full covariance matrix. The cell e.s.d.'s are taken into account individually in the estimation of e.s.d.'s in distances, angles and torsion angles; correlations between e.s.d.'s in cell parameters are only used when they are defined by crystal symmetry. An approximate (isotropic) treatment of cell e.s.d.'s is used for estimating e.s.d.'s involving l.s. planes.
Refinement. Refinement of *F*^2^ against ALL reflections. The weighted *R*-factor *wR* and goodness of fit *S* are based on *F*^2^, conventional *R*-factors *R* are based on *F*, with *F* set to zero for negative *F*^2^. The threshold expression of *F*^2^ > σ(*F*^2^) is used only for calculating *R*-factors(gt) *etc*. and is not relevant to the choice of reflections for refinement. *R*-factors based on *F*^2^ are statistically about twice as large as those based on *F*, and *R*- factors based on ALL data will be even larger.

### Fractional atomic coordinates and isotropic or equivalent isotropic displacement parameters (Å^2^)

**Table:**

	*x*	*y*	*z*	*U*_iso_*/*U*_eq_	
Ru1	0.859197 (11)	0.28327 (4)	0.574586 (17)	0.01982 (13)	
Cl1	0.91585 (4)	0.08062 (14)	0.60413 (6)	0.0281 (3)	
Cl2	0.82324 (4)	0.09300 (16)	0.49193 (6)	0.0355 (3)	
N1	0.91793 (11)	0.3570 (4)	0.46538 (17)	0.0191 (7)	
N2	0.91101 (12)	0.5520 (4)	0.53600 (17)	0.0214 (8)	
C1	0.89796 (14)	0.3939 (5)	0.5173 (2)	0.0201 (9)	
C2	0.94310 (13)	0.4900 (5)	0.4509 (2)	0.0201 (9)	
C3	0.96901 (14)	0.5119 (6)	0.4034 (2)	0.0241 (10)	
H3A	0.9716	0.4287	0.3716	0.029*	
C4	0.99086 (14)	0.6595 (6)	0.4043 (2)	0.0262 (10)	
H4A	1.0093	0.6778	0.3728	0.031*	
C5	0.98659 (14)	0.7840 (6)	0.4505 (2)	0.0252 (10)	
H5A	1.0026	0.8834	0.4500	0.030*	
C6	0.95983 (14)	0.7658 (6)	0.4967 (2)	0.0238 (10)	
H6A	0.9562	0.8509	0.5271	0.029*	
C7	0.93834 (13)	0.6142 (5)	0.4960 (2)	0.0205 (9)	
C8	0.89772 (16)	0.6340 (6)	0.5930 (2)	0.0276 (10)	
H8A	0.8851	0.7437	0.5802	0.033*	
H8B	0.9228	0.6485	0.6293	0.033*	
C9	0.86420 (15)	0.5220 (6)	0.6150 (2)	0.0242 (10)	
C10	0.87528 (15)	0.4087 (6)	0.6688 (2)	0.0249 (10)	
C11	0.84433 (15)	0.2836 (6)	0.6779 (2)	0.0265 (10)	
H11A	0.8549	0.1881	0.7073	0.032*	
C12	0.80490 (15)	0.2661 (6)	0.6353 (2)	0.0274 (10)	
C13	0.79482 (15)	0.3822 (6)	0.5822 (2)	0.0292 (11)	
H13A	0.7701	0.3561	0.5457	0.035*	
C14	0.82217 (15)	0.5141 (6)	0.5727 (2)	0.0277 (10)	
C15	0.80849 (18)	0.6362 (7)	0.5183 (3)	0.0381 (12)	
H15A	0.7922	0.7258	0.5346	0.057*	
H15B	0.7901	0.5812	0.4810	0.057*	
H15C	0.8342	0.6816	0.5036	0.057*	
C16	0.91763 (16)	0.4145 (6)	0.7169 (2)	0.0330 (11)	
H16A	0.9142	0.4825	0.7553	0.049*	
H16B	0.9401	0.4624	0.6950	0.049*	
H16C	0.9261	0.3024	0.7318	0.049*	
C17	0.77421 (17)	0.1265 (7)	0.6407 (3)	0.0396 (13)	
H17A	0.7546	0.1577	0.6710	0.059*	
H17B	0.7907	0.0282	0.6578	0.059*	
H17C	0.7573	0.1024	0.5968	0.059*	
C18	0.91506 (14)	0.2017 (5)	0.4291 (2)	0.0203 (9)	
H18A	0.8997	0.1196	0.4523	0.024*	
H18B	0.9446	0.1596	0.4287	0.024*	
C19	0.89144 (14)	0.2196 (5)	0.3583 (2)	0.0212 (9)	
C20	0.85252 (15)	0.3085 (6)	0.3461 (2)	0.0264 (10)	
H20A	0.8427	0.3619	0.3820	0.032*	
C21	0.82819 (16)	0.3203 (6)	0.2835 (2)	0.0320 (11)	
H21A	0.8023	0.3838	0.2762	0.038*	
C22	0.84158 (18)	0.2394 (6)	0.2313 (2)	0.0339 (12)	
H22A	0.8243	0.2426	0.1884	0.041*	
C23	0.88025 (16)	0.1539 (6)	0.2418 (2)	0.0296 (11)	
H23A	0.8896	0.1011	0.2054	0.036*	
C24	0.90593 (16)	0.1429 (6)	0.3047 (2)	0.0262 (10)	
C25	0.94799 (17)	0.0499 (7)	0.3128 (3)	0.0372 (12)	
H25A	0.9520	0.0060	0.2697	0.056*	
H25B	0.9474	−0.0415	0.3439	0.056*	
H25C	0.9720	0.1244	0.3299	0.056*	
C26	0.81998 (16)	0.8842 (7)	0.3454 (3)	0.0353 (12)	
H26A	0.8182	0.9577	0.3835	0.042*	
H26B	0.8224	0.9543	0.3066	0.042*	
Cl3	0.77287 (6)	0.7648 (3)	0.32788 (10)	0.0729 (6)	
Cl4	0.86627 (5)	0.7586 (2)	0.36431 (8)	0.0496 (4)	

### Atomic displacement parameters (Å^2^)

**Table:**

	*U*^11^	*U*^22^	*U*^33^	*U*^12^	*U*^13^	*U*^23^
Ru1	0.0249 (2)	0.0163 (2)	0.0197 (2)	−0.00305 (13)	0.00788 (14)	−0.00191 (13)
Cl1	0.0343 (6)	0.0203 (6)	0.0332 (6)	0.0028 (4)	0.0156 (5)	0.0037 (5)
Cl2	0.0372 (7)	0.0366 (7)	0.0346 (7)	−0.0150 (5)	0.0119 (5)	−0.0159 (5)
N1	0.0201 (18)	0.0165 (18)	0.0207 (18)	−0.0012 (14)	0.0035 (14)	−0.0015 (15)
N2	0.031 (2)	0.0133 (18)	0.0213 (19)	−0.0040 (15)	0.0094 (15)	−0.0024 (14)
C1	0.026 (2)	0.017 (2)	0.017 (2)	−0.0001 (17)	0.0010 (17)	0.0010 (17)
C2	0.021 (2)	0.017 (2)	0.022 (2)	−0.0026 (17)	0.0024 (17)	0.0032 (17)
C3	0.027 (2)	0.023 (2)	0.023 (2)	0.0029 (18)	0.0048 (18)	0.0029 (18)
C4	0.023 (2)	0.028 (3)	0.030 (2)	−0.0005 (19)	0.0099 (19)	0.010 (2)
C5	0.022 (2)	0.020 (2)	0.033 (3)	−0.0061 (18)	0.0033 (19)	0.0048 (19)
C6	0.023 (2)	0.021 (2)	0.026 (2)	−0.0019 (18)	0.0015 (18)	0.0011 (19)
C7	0.021 (2)	0.019 (2)	0.021 (2)	−0.0009 (17)	0.0037 (17)	0.0026 (17)
C8	0.039 (3)	0.020 (2)	0.026 (2)	−0.006 (2)	0.013 (2)	−0.0064 (19)
C9	0.031 (2)	0.020 (2)	0.023 (2)	0.0011 (19)	0.0112 (19)	−0.0055 (18)
C10	0.032 (2)	0.022 (2)	0.023 (2)	0.0023 (19)	0.0114 (19)	−0.0065 (18)
C11	0.033 (3)	0.021 (2)	0.027 (2)	0.0008 (19)	0.012 (2)	−0.0024 (19)
C12	0.029 (2)	0.029 (3)	0.029 (3)	−0.002 (2)	0.017 (2)	−0.004 (2)
C13	0.029 (2)	0.031 (3)	0.028 (2)	0.006 (2)	0.008 (2)	−0.005 (2)
C14	0.030 (2)	0.024 (2)	0.031 (3)	0.0044 (19)	0.012 (2)	−0.003 (2)
C15	0.042 (3)	0.031 (3)	0.039 (3)	0.005 (2)	0.000 (2)	0.005 (2)
C16	0.038 (3)	0.031 (3)	0.030 (3)	−0.002 (2)	0.007 (2)	−0.003 (2)
C17	0.038 (3)	0.035 (3)	0.049 (3)	−0.010 (2)	0.017 (2)	−0.001 (2)
C18	0.024 (2)	0.016 (2)	0.022 (2)	−0.0007 (17)	0.0056 (17)	−0.0036 (17)
C19	0.029 (2)	0.015 (2)	0.020 (2)	−0.0061 (17)	0.0062 (18)	−0.0014 (17)
C20	0.031 (2)	0.024 (2)	0.024 (2)	−0.0016 (19)	0.0067 (19)	−0.0007 (19)
C21	0.030 (3)	0.034 (3)	0.031 (3)	−0.003 (2)	0.003 (2)	0.005 (2)
C22	0.044 (3)	0.034 (3)	0.022 (3)	−0.012 (2)	0.002 (2)	0.005 (2)
C23	0.047 (3)	0.025 (3)	0.019 (2)	−0.007 (2)	0.012 (2)	−0.0028 (19)
C24	0.039 (3)	0.018 (2)	0.023 (2)	−0.004 (2)	0.010 (2)	0.0002 (18)
C25	0.049 (3)	0.032 (3)	0.033 (3)	0.005 (2)	0.013 (2)	−0.004 (2)
C26	0.041 (3)	0.034 (3)	0.030 (3)	−0.001 (2)	0.003 (2)	−0.003 (2)
Cl3	0.0424 (9)	0.0820 (13)	0.0869 (13)	−0.0229 (9)	−0.0094 (9)	0.0202 (10)
Cl4	0.0440 (8)	0.0547 (9)	0.0488 (8)	0.0042 (7)	0.0051 (6)	0.0140 (7)

### Geometric parameters (Å, °)

**Table:**

Ru1—C1	2.039 (4)	C12—C17	1.502 (7)
Ru1—C9	2.099 (4)	C13—C14	1.405 (7)
Ru1—C10	2.162 (4)	C13—H13A	1.0000
Ru1—C14	2.198 (5)	C14—C15	1.496 (7)
Ru1—C13	2.203 (5)	C15—H15A	0.9800
Ru1—C11	2.246 (5)	C15—H15B	0.9800
Ru1—C12	2.282 (5)	C15—H15C	0.9800
Ru1—Cl1	2.4167 (12)	C16—H16A	0.9800
Ru1—Cl2	2.4175 (13)	C16—H16B	0.9800
N1—C1	1.359 (6)	C16—H16C	0.9800
N1—C2	1.398 (5)	C17—H17A	0.9800
N1—C18	1.456 (5)	C17—H17B	0.9800
N2—C1	1.378 (5)	C17—H17C	0.9800
N2—C7	1.381 (6)	C18—C19	1.515 (6)
N2—C8	1.466 (6)	C18—H18A	0.9900
C2—C3	1.384 (6)	C18—H18B	0.9900
C2—C7	1.392 (6)	C19—C20	1.401 (6)
C3—C4	1.377 (6)	C19—C24	1.405 (6)
C3—H3A	0.9500	C20—C21	1.377 (6)
C4—C5	1.407 (7)	C20—H20A	0.9500
C4—H4A	0.9500	C21—C22	1.380 (8)
C5—C6	1.378 (7)	C21—H21A	0.9500
C5—H5A	0.9500	C22—C23	1.380 (7)
C6—C7	1.399 (6)	C22—H22A	0.9500
C6—H6A	0.9500	C23—C24	1.397 (7)
C8—C9	1.515 (6)	C23—H23A	0.9500
C8—H8A	0.9900	C24—C25	1.503 (7)
C8—H8B	0.9900	C25—H25A	0.9800
C9—C10	1.430 (6)	C25—H25B	0.9800
C9—C14	1.446 (6)	C25—H25C	0.9800
C10—C11	1.438 (6)	C26—Cl3	1.750 (5)
C10—C16	1.509 (7)	C26—Cl4	1.760 (5)
C11—C12	1.390 (7)	C26—H26A	0.9900
C11—H11A	1.0000	C26—H26B	0.9900
C12—C13	1.430 (7)		
			
C1—Ru1—C9	79.10 (17)	C16—C10—Ru1	129.7 (3)
C1—Ru1—C10	103.77 (17)	C12—C11—C10	122.4 (4)
C9—Ru1—C10	39.19 (17)	C12—C11—Ru1	73.5 (3)
C1—Ru1—C14	89.00 (17)	C10—C11—Ru1	67.8 (2)
C9—Ru1—C14	39.24 (17)	C12—C11—H11A	117.6
C10—Ru1—C14	69.83 (17)	C10—C11—H11A	117.6
C1—Ru1—C13	121.82 (18)	Ru1—C11—H11A	117.6
C9—Ru1—C13	69.08 (18)	C11—C12—C13	117.7 (4)
C10—Ru1—C13	80.71 (18)	C11—C12—C17	122.8 (5)
C14—Ru1—C13	37.24 (18)	C13—C12—C17	119.5 (4)
C1—Ru1—C11	141.73 (17)	C11—C12—Ru1	70.7 (3)
C9—Ru1—C11	68.95 (17)	C13—C12—Ru1	68.4 (3)
C10—Ru1—C11	38.02 (17)	C17—C12—Ru1	129.4 (3)
C14—Ru1—C11	79.57 (17)	C14—C13—C12	123.1 (4)
C13—Ru1—C11	65.68 (18)	C14—C13—Ru1	71.2 (3)
C1—Ru1—C12	156.54 (17)	C12—C13—Ru1	74.4 (3)
C9—Ru1—C12	81.52 (17)	C14—C13—H13A	117.9
C10—Ru1—C12	67.76 (17)	C12—C13—H13A	117.9
C14—Ru1—C12	67.59 (17)	Ru1—C13—H13A	117.9
C13—Ru1—C12	37.12 (18)	C13—C14—C9	117.7 (4)
C11—Ru1—C12	35.75 (17)	C13—C14—C15	120.3 (4)
C1—Ru1—Cl1	87.51 (12)	C9—C14—C15	121.9 (4)
C9—Ru1—Cl1	121.77 (13)	C13—C14—Ru1	71.6 (3)
C10—Ru1—Cl1	92.85 (13)	C9—C14—Ru1	66.7 (2)
C14—Ru1—Cl1	160.95 (13)	C15—C14—Ru1	131.1 (3)
C13—Ru1—Cl1	150.67 (13)	C14—C15—H15A	109.5
C11—Ru1—Cl1	91.71 (13)	C14—C15—H15B	109.5
C12—Ru1—Cl1	114.09 (13)	H15A—C15—H15B	109.5
C1—Ru1—Cl2	97.42 (12)	C14—C15—H15C	109.5
C9—Ru1—Cl2	149.00 (13)	H15A—C15—H15C	109.5
C10—Ru1—Cl2	158.81 (13)	H15B—C15—H15C	109.5
C14—Ru1—Cl2	110.51 (13)	C10—C16—H16A	109.5
C13—Ru1—Cl2	87.77 (13)	C10—C16—H16B	109.5
C11—Ru1—Cl2	120.82 (12)	H16A—C16—H16B	109.5
C12—Ru1—Cl2	92.40 (12)	C10—C16—H16C	109.5
Cl1—Ru1—Cl2	88.52 (5)	H16A—C16—H16C	109.5
C1—N1—C2	110.6 (3)	H16B—C16—H16C	109.5
C1—N1—C18	126.4 (4)	C12—C17—H17A	109.5
C2—N1—C18	123.0 (3)	C12—C17—H17B	109.5
C1—N2—C7	111.1 (4)	H17A—C17—H17B	109.5
C1—N2—C8	122.0 (4)	C12—C17—H17C	109.5
C7—N2—C8	126.9 (4)	H17A—C17—H17C	109.5
N1—C1—N2	105.5 (4)	H17B—C17—H17C	109.5
N1—C1—Ru1	139.2 (3)	N1—C18—C19	112.5 (3)
N2—C1—Ru1	115.2 (3)	N1—C18—H18A	109.1
C3—C2—C7	121.3 (4)	C19—C18—H18A	109.1
C3—C2—N1	132.1 (4)	N1—C18—H18B	109.1
C7—C2—N1	106.6 (4)	C19—C18—H18B	109.1
C4—C3—C2	117.0 (4)	H18A—C18—H18B	107.8
C4—C3—H3A	121.5	C20—C19—C24	118.6 (4)
C2—C3—H3A	121.5	C20—C19—C18	118.9 (4)
C3—C4—C5	121.7 (4)	C24—C19—C18	122.4 (4)
C3—C4—H4A	119.1	C21—C20—C19	121.7 (4)
C5—C4—H4A	119.1	C21—C20—H20A	119.2
C6—C5—C4	121.8 (4)	C19—C20—H20A	119.2
C6—C5—H5A	119.1	C20—C21—C22	119.6 (5)
C4—C5—H5A	119.1	C20—C21—H21A	120.2
C5—C6—C7	115.9 (4)	C22—C21—H21A	120.2
C5—C6—H6A	122.0	C21—C22—C23	119.7 (5)
C7—C6—H6A	122.0	C21—C22—H22A	120.1
N2—C7—C2	106.3 (4)	C23—C22—H22A	120.1
N2—C7—C6	131.5 (4)	C22—C23—C24	121.6 (4)
C2—C7—C6	122.2 (4)	C22—C23—H23A	119.2
N2—C8—C9	105.9 (4)	C24—C23—H23A	119.2
N2—C8—H8A	110.6	C23—C24—C19	118.7 (4)
C9—C8—H8A	110.6	C23—C24—C25	119.1 (4)
N2—C8—H8B	110.6	C19—C24—C25	122.3 (4)
C9—C8—H8B	110.6	C24—C25—H25A	109.5
H8A—C8—H8B	108.7	C24—C25—H25B	109.5
C10—C9—C14	120.4 (4)	H25A—C25—H25B	109.5
C10—C9—C8	121.7 (4)	C24—C25—H25C	109.5
C14—C9—C8	117.1 (4)	H25A—C25—H25C	109.5
C10—C9—Ru1	72.8 (3)	H25B—C25—H25C	109.5
C14—C9—Ru1	74.1 (3)	Cl3—C26—Cl4	111.1 (3)
C8—C9—Ru1	116.3 (3)	Cl3—C26—H26A	109.4
C9—C10—C11	118.3 (4)	Cl4—C26—H26A	109.4
C9—C10—C16	123.4 (4)	Cl3—C26—H26B	109.4
C11—C10—C16	118.3 (4)	Cl4—C26—H26B	109.4
C9—C10—Ru1	68.0 (2)	H26A—C26—H26B	108.0
C11—C10—Ru1	74.2 (3)		
			
C2—N1—C1—N2	−0.4 (4)	C14—Ru1—C11—C12	65.9 (3)
C18—N1—C1—N2	178.7 (4)	C13—Ru1—C11—C12	29.6 (3)
C2—N1—C1—Ru1	−176.3 (4)	Cl1—Ru1—C11—C12	−131.1 (3)
C18—N1—C1—Ru1	2.8 (7)	Cl2—Ru1—C11—C12	−41.8 (3)
C7—N2—C1—N1	0.7 (5)	C1—Ru1—C11—C10	4.3 (4)
C8—N2—C1—N1	−177.2 (4)	C9—Ru1—C11—C10	−31.1 (3)
C7—N2—C1—Ru1	177.7 (3)	C14—Ru1—C11—C10	−70.5 (3)
C8—N2—C1—Ru1	−0.1 (5)	C13—Ru1—C11—C10	−106.9 (3)
C9—Ru1—C1—N1	−178.4 (5)	C12—Ru1—C11—C10	−136.4 (4)
C10—Ru1—C1—N1	150.9 (5)	Cl1—Ru1—C11—C10	92.4 (3)
C14—Ru1—C1—N1	−140.1 (5)	Cl2—Ru1—C11—C10	−178.3 (2)
C13—Ru1—C1—N1	−121.6 (5)	C10—C11—C12—C13	−2.4 (7)
C11—Ru1—C1—N1	148.2 (4)	Ru1—C11—C12—C13	−51.5 (4)
C12—Ru1—C1—N1	−143.5 (5)	C10—C11—C12—C17	174.2 (4)
Cl1—Ru1—C1—N1	58.6 (5)	Ru1—C11—C12—C17	125.1 (5)
Cl2—Ru1—C1—N1	−29.6 (5)	C10—C11—C12—Ru1	49.1 (4)
C9—Ru1—C1—N2	6.0 (3)	C1—Ru1—C12—C11	−100.0 (5)
C10—Ru1—C1—N2	−24.7 (3)	C9—Ru1—C12—C11	−65.5 (3)
C14—Ru1—C1—N2	44.2 (3)	C10—Ru1—C12—C11	−27.3 (3)
C13—Ru1—C1—N2	62.8 (4)	C14—Ru1—C12—C11	−103.7 (3)
C11—Ru1—C1—N2	−27.4 (5)	C13—Ru1—C12—C11	−131.9 (4)
C12—Ru1—C1—N2	40.8 (6)	Cl1—Ru1—C12—C11	55.6 (3)
Cl1—Ru1—C1—N2	−117.0 (3)	Cl2—Ru1—C12—C11	145.0 (3)
Cl2—Ru1—C1—N2	154.8 (3)	C1—Ru1—C12—C13	31.8 (6)
C1—N1—C2—C3	180.0 (4)	C9—Ru1—C12—C13	66.3 (3)
C18—N1—C2—C3	0.9 (7)	C10—Ru1—C12—C13	104.6 (3)
C1—N1—C2—C7	0.0 (5)	C14—Ru1—C12—C13	28.1 (3)
C18—N1—C2—C7	−179.1 (4)	C11—Ru1—C12—C13	131.9 (4)
C7—C2—C3—C4	2.1 (6)	Cl1—Ru1—C12—C13	−172.6 (2)
N1—C2—C3—C4	−177.9 (4)	Cl2—Ru1—C12—C13	−83.1 (3)
C2—C3—C4—C5	−1.0 (6)	C1—Ru1—C12—C17	142.9 (5)
C3—C4—C5—C6	−1.0 (7)	C9—Ru1—C12—C17	177.4 (5)
C4—C5—C6—C7	2.0 (6)	C10—Ru1—C12—C17	−144.3 (5)
C1—N2—C7—C2	−0.7 (5)	C14—Ru1—C12—C17	139.2 (5)
C8—N2—C7—C2	177.1 (4)	C13—Ru1—C12—C17	111.1 (6)
C1—N2—C7—C6	−179.0 (4)	C11—Ru1—C12—C17	−117.0 (6)
C8—N2—C7—C6	−1.2 (8)	Cl1—Ru1—C12—C17	−61.5 (5)
C3—C2—C7—N2	−179.6 (4)	Cl2—Ru1—C12—C17	28.0 (5)
N1—C2—C7—N2	0.4 (4)	C11—C12—C13—C14	−1.9 (7)
C3—C2—C7—C6	−1.1 (6)	C17—C12—C13—C14	−178.5 (4)
N1—C2—C7—C6	178.9 (4)	Ru1—C12—C13—C14	−54.4 (4)
C5—C6—C7—N2	177.1 (4)	C11—C12—C13—Ru1	52.5 (4)
C5—C6—C7—C2	−0.9 (6)	C17—C12—C13—Ru1	−124.1 (4)
C1—N2—C8—C9	−8.3 (6)	C1—Ru1—C13—C14	−31.7 (3)
C7—N2—C8—C9	174.2 (4)	C9—Ru1—C13—C14	29.9 (3)
N2—C8—C9—C10	98.1 (5)	C10—Ru1—C13—C14	68.8 (3)
N2—C8—C9—C14	−71.9 (5)	C11—Ru1—C13—C14	105.4 (3)
N2—C8—C9—Ru1	12.9 (5)	C12—Ru1—C13—C14	134.0 (4)
C1—Ru1—C9—C10	−128.3 (3)	Cl1—Ru1—C13—C14	147.9 (2)
C14—Ru1—C9—C10	129.7 (4)	Cl2—Ru1—C13—C14	−129.1 (3)
C13—Ru1—C9—C10	101.3 (3)	C1—Ru1—C13—C12	−165.7 (3)
C11—Ru1—C9—C10	30.3 (3)	C9—Ru1—C13—C12	−104.1 (3)
C12—Ru1—C9—C10	65.0 (3)	C10—Ru1—C13—C12	−65.2 (3)
Cl1—Ru1—C9—C10	−48.2 (3)	C14—Ru1—C13—C12	−134.0 (4)
Cl2—Ru1—C9—C10	145.4 (2)	C11—Ru1—C13—C12	−28.5 (3)
C1—Ru1—C9—C14	102.0 (3)	Cl1—Ru1—C13—C12	13.9 (4)
C10—Ru1—C9—C14	−129.7 (4)	Cl2—Ru1—C13—C12	96.9 (3)
C13—Ru1—C9—C14	−28.4 (3)	C12—C13—C14—C9	6.2 (7)
C11—Ru1—C9—C14	−99.5 (3)	Ru1—C13—C14—C9	−49.7 (4)
C12—Ru1—C9—C14	−64.7 (3)	C12—C13—C14—C15	−176.7 (4)
Cl1—Ru1—C9—C14	−177.9 (2)	Ru1—C13—C14—C15	127.4 (4)
Cl2—Ru1—C9—C14	15.7 (4)	C12—C13—C14—Ru1	55.9 (4)
C1—Ru1—C9—C8	−10.9 (3)	C10—C9—C14—C13	−6.4 (6)
C10—Ru1—C9—C8	117.4 (5)	C8—C9—C14—C13	163.8 (4)
C14—Ru1—C9—C8	−112.8 (4)	Ru1—C9—C14—C13	52.0 (4)
C13—Ru1—C9—C8	−141.3 (4)	C10—C9—C14—C15	176.6 (4)
C11—Ru1—C9—C8	147.7 (4)	C8—C9—C14—C15	−13.3 (6)
C12—Ru1—C9—C8	−177.6 (4)	Ru1—C9—C14—C15	−125.0 (4)
Cl1—Ru1—C9—C8	69.3 (4)	C10—C9—C14—Ru1	−58.4 (4)
Cl2—Ru1—C9—C8	−97.1 (4)	C8—C9—C14—Ru1	111.8 (4)
C14—C9—C10—C11	2.5 (6)	C1—Ru1—C14—C13	153.4 (3)
C8—C9—C10—C11	−167.2 (4)	C9—Ru1—C14—C13	−132.7 (4)
Ru1—C9—C10—C11	−56.5 (4)	C10—Ru1—C14—C13	−101.5 (3)
C14—C9—C10—C16	−177.0 (4)	C11—Ru1—C14—C13	−63.3 (3)
C8—C9—C10—C16	13.3 (7)	C12—Ru1—C14—C13	−28.0 (3)
Ru1—C9—C10—C16	124.0 (4)	Cl1—Ru1—C14—C13	−127.2 (4)
C14—C9—C10—Ru1	59.0 (4)	Cl2—Ru1—C14—C13	55.9 (3)
C8—C9—C10—Ru1	−110.7 (4)	C1—Ru1—C14—C9	−73.9 (3)
C1—Ru1—C10—C9	52.5 (3)	C10—Ru1—C14—C9	31.2 (3)
C14—Ru1—C10—C9	−31.2 (3)	C13—Ru1—C14—C9	132.7 (4)
C13—Ru1—C10—C9	−68.2 (3)	C11—Ru1—C14—C9	69.4 (3)
C11—Ru1—C10—C9	−130.2 (4)	C12—Ru1—C14—C9	104.6 (3)
C12—Ru1—C10—C9	−104.4 (3)	Cl1—Ru1—C14—C9	5.5 (5)
Cl1—Ru1—C10—C9	140.6 (2)	Cl2—Ru1—C14—C9	−171.4 (2)
Cl2—Ru1—C10—C9	−126.1 (3)	C1—Ru1—C14—C15	39.0 (5)
C1—Ru1—C10—C11	−177.3 (3)	C9—Ru1—C14—C15	112.8 (6)
C9—Ru1—C10—C11	130.2 (4)	C10—Ru1—C14—C15	144.0 (5)
C14—Ru1—C10—C11	99.0 (3)	C13—Ru1—C14—C15	−114.5 (6)
C13—Ru1—C10—C11	62.1 (3)	C11—Ru1—C14—C15	−177.8 (5)
C12—Ru1—C10—C11	25.8 (3)	C12—Ru1—C14—C15	−142.5 (5)
Cl1—Ru1—C10—C11	−89.1 (3)	Cl1—Ru1—C14—C15	118.4 (5)
Cl2—Ru1—C10—C11	4.1 (5)	Cl2—Ru1—C14—C15	−58.6 (5)
C1—Ru1—C10—C16	−63.4 (4)	C1—N1—C18—C19	112.6 (5)
C9—Ru1—C10—C16	−115.9 (5)	C2—N1—C18—C19	−68.4 (5)
C14—Ru1—C10—C16	−147.1 (5)	N1—C18—C19—C20	−45.8 (5)
C13—Ru1—C10—C16	175.9 (5)	N1—C18—C19—C24	137.6 (4)
C11—Ru1—C10—C16	113.9 (5)	C24—C19—C20—C21	0.8 (7)
C12—Ru1—C10—C16	139.7 (5)	C18—C19—C20—C21	−175.9 (4)
Cl1—Ru1—C10—C16	24.7 (4)	C19—C20—C21—C22	1.7 (7)
Cl2—Ru1—C10—C16	118.0 (4)	C20—C21—C22—C23	−3.0 (8)
C9—C10—C11—C12	2.0 (7)	C21—C22—C23—C24	1.7 (7)
C16—C10—C11—C12	−178.5 (4)	C22—C23—C24—C19	0.9 (7)
Ru1—C10—C11—C12	−51.5 (4)	C22—C23—C24—C25	−179.2 (5)
C9—C10—C11—Ru1	53.5 (3)	C20—C19—C24—C23	−2.1 (6)
C16—C10—C11—Ru1	−127.0 (4)	C18—C19—C24—C23	174.5 (4)
C1—Ru1—C11—C12	140.7 (3)	C20—C19—C24—C25	177.9 (4)
C9—Ru1—C11—C12	105.3 (3)	C18—C19—C24—C25	−5.5 (7)
C10—Ru1—C11—C12	136.4 (4)		

### Hydrogen-bond geometry (Å, °)

**Table:**

*D*—H···*A*	*D*—H	H···*A*	*D*···*A*	*D*—H···*A*
C15—H15C···N2	0.98	2.60	3.244 (7)	123
C18—H18A···Cl2	0.99	2.67	3.468 (5)	138
C23—H23A···Cl1^i^	0.95	2.78	3.730 (5)	175
C26—H26A···Cl2^ii^	0.99	2.46	3.431 (6)	168

Symmetry codes: (i) *x*, −*y*, *z*−1/2; (ii) *x*, *y*+1, *z*.
